# The value of FOLFIRINOX in advanced pancreatic cancer: balancing efficacy, toxicity, and quality of life

**DOI:** 10.1007/s00520-026-10568-3

**Published:** 2026-03-26

**Authors:** Gaby J. Strijk, Aniek E. van Diepen, Johanna W. Wilmink, Judith A. C. Rietjens, Brigitte C. M. Haberkorn, Anne M. Stiggelbout, Marjolein Y. V. Homs, Judith de Vos-Geelen, Casper H. J. van Eijck, Casper W. F. van Eijck

**Affiliations:** 1 Solid Tumour Immunology Research Rotterdam (STIRR) Group, Department of Pulmonary Medicine, Erasmus University Medical Centre, Rotterdam, The Netherlands; 2 Department of Internal Medicine, Division of Medical Oncology, GROW Research Institute for Oncology & Reproduction, Maastricht UMC+, Maastricht, the Netherlands; 3 Department of Medical Oncology, Amsterdam University Medical Centre, Amsterdam, The Netherlands; 4 Department of Public Health, Erasmus University Medical Centre, Rotterdam, The Netherlands; 5 Department of Design, Organisation and Strategy, Faculty of Industrial Design Engineering, Delft University of Technology, Delft, The Netherlands; 6 Department of Medical Oncology, Maasstad Hospital, Rotterdam, The Netherlands; 7 Medical Decision Making, Department of Biomedical Data Sciences, Leiden University Medical Centre, Leiden, The Netherlands; 8 Department of Medical Oncology, Erasmus University Medical Centre, Rotterdam, The Netherlands

**Keywords:** Ethics, Medical, Maintenance chemotherapy, Palliative care, Pancreatic neoplasms, Patient-centred care, Treatment outcome

## Abstract

**Purpose:**

Balancing therapeutic benefit and treatment burden remains a major challenge in administering FOLFIRINOX for advanced pancreatic ductal adenocarcinoma (PDAC). More than 60% of patients experience grade ≥ 3 adverse events (AEs), and only about 30% achieve a tumour response. This study examines the value of FOLFIRINOX chemotherapy by contextualising treatment efficacy, toxicity, and durability of response within ethical, financial, and person-centred frameworks. The aim is to support shared decision-making and optimise clinical outcomes and quality of life.

**Methods:**

Patients with advanced PDAC receiving palliative FOLFIRINOX were included. Radiologic response was assessed using RECIST 1.1 at sequential time points during and after therapy. Patients were classified as having either progressive disease or disease control, the latter encompassing complete response, partial response, and stable disease.

**Results:**

Amongst 84 advanced PDAC patients, 14 (17%) discontinued FOLFIRINOX before completing four cycles due to grade ≥ 3 AEs. Another four patients (5%) terminated FOLFIRINOX treatment after four cycles despite exhibiting disease control. Overall, 36 (43%) patients had at least one FOLFIRINOX-related grade ≥ 3 AE. Amongst the 66 patients who completed ≥ 4 cycles, 52 (79%) achieved disease control at FOLFIRINOX completion. However, disease control declined rapidly after therapy, falling to 41% at 6 months, 21% at 1 year, and 11% at 2 years. LAPC patients achieved disease control more frequently than patients with metastatic disease. Nevertheless, both groups experienced steep declines in disease control over time, from 85% and 69% at treatment completion to 32% and 4% at 1 year in LAPC and metastatic PDAC, respectively. Overall, 36 patients (55%) received subsequent systemic or locoregional therapies, most commonly gemcitabine/nab-paclitaxel (6%), SBRT (17%), or immunotherapy combined with SBRT (11%).

**Conclusion:**

Whilst FOLFIRINOX achieves high initial disease control in advanced PDAC, its benefit is limited by substantial toxicity and poor durability. Only a minority of patients maintain disease control beyond 6 months, which is strongly associated with improved survival. These findings suggest a need to redefine ‘valuable FOLFIRINOX chemotherapy’ to encompass not only initial response but also sustained disease control, toxicity management, and shared decision-making to optimise the benefit of FOLFIRINOX for patients.

**Supplementary Information:**

The online version contains supplementary material available at 10.1007/s00520-026-10568-3.

## Introduction

Pancreatic ductal adenocarcinoma (PDAC) is amongst the most lethal malignancies, characterised by its aggressive progression and frequent late-stage diagnosis. With a 5-year survival rate of just 8%, PDAC holds the lowest survival rate amongst all cancers in Europe [1]. Between 1990 and 2016, pancreatic cancer accounted for the highest increase in mortality amongst the top five cancer-related causes of death in Europe [2].

PDAC, like many cancers, presents a profound and multifaceted challenge to modern healthcare, affecting numerous lives and placing increasing pressure on healthcare systems. This has led to rising demand for intensive treatments, such as surgery, chemotherapy, radiotherapy, and immunotherapy. However, despite their therapeutic potential, these treatments often impose substantial physical, emotional, and financial burdens. Consequently, although cancer care costs represent a relatively small share of total healthcare expenditure compared to other chronic diseases, they are rapidly escalating and must be carefully considered in policy and clinical decisions [3, 4].

At the individual level, an essential dilemma in PDAC treatment lies in balancing therapeutic efficacy with toxicity. Whilst adverse events (AEs) may be acceptable when a tumour demonstrates a meaningful response, i.e., shrinkage or disease stabilisation, side effects can sometimes become intolerable and impair patients’ quality of life (QoL). In some cases, toxicity becomes intolerable and requires treatment discontinuation. Paradoxically, severe, or even life-threatening, toxicities may also occur in patients whose tumours initially respond. Conversely, many patients derive little or no benefit from those therapies, experiencing disease progression whilst enduring substantial treatment-related morbidity [5–7].

Given these therapeutic challenges, treatment strategies for PDAC remain largely determined by disease stage and patient condition. Surgical resection, typically combined with (neo)adjuvant chemotherapy or chemoradiotherapy, remains the only curative option. However, fewer than 20% of individuals with PDAC are eligible for surgery due to advanced disease at diagnosis [7]. For the vast majority, palliative chemotherapy remains the mainstay of treatment in advanced disease, aiming to extend life and alleviate symptoms such as pain, jaundice, and gastrointestinal obstruction. Standard palliative regimens include gemcitabine-based therapies and the more intensive FOLFIRINOX combination (folinic acid, 5-fluorouracil, irinotecan, and oxaliplatin) [6, 8, 9].

FOLFIRINOX is amongst the most active regimens in PDAC and can induce substantial initial disease control, but its benefit appears less consistent than initially assumed: recent randomised trials have not demonstrated a clear overall survival advantage over gemcitabine/nab-paclitaxel and, in some settings, even suggest a trend towards inferior outcomes. At the same time, the toxicity burden remains considerable, with most patients experiencing grade ≥ 3 adverse events, and certain toxicities (such as grade 3–4 anorexia) occurring more frequently than with gemcitabine/nab-paclitaxel [5, 8–11]. As a result, many patients endure considerable treatment-related burden with limited to no therapeutic gain. Complicating matters, radiologic assessments performed immediately after FOLFIRINOX chemotherapy may show tumour shrinkage, only for rapid progression to follow shortly after chemotherapy cessation. This concern is reinforced by recent randomised trials in both metastatic and locally advanced PDAC, which increasingly fail to demonstrate superiority of (modified) FOLFIRINOX over alternative regimens such as gemcitabine/nab-paclitaxel. These patterns underscore the urgent need to define what constitutes ‘valuable’ FOLFIRINOX chemotherapy, meaning a treatment course in which the substantial burden is justified not only by tumour response but by sustained survival, symptom relief, and preservation of quality of life.

This study aims to evaluate the short- and long-term effectiveness of FOLFIRINOX in patients with advanced PDAC by assessing treatment response and survival outcomes immediately after therapy and at 6 months, 1 year, and 2 years post-treatment. These clinical data are interpreted through a broader lens, incorporating ethical, financial, and practical considerations drawn from the literature. Ultimately, this work seeks to foster deeper dialogue around the risks and benefits of FOLFIRINOX chemotherapy, promoting more person-centred care, shared decision-making, and improved outcomes for individuals with PDAC.

## Material and methods

### Advanced PDAC cohort and clinical procedures

This study included patients with advanced PDAC, encompassing both locally advanced pancreatic cancer (LAPC) and synchronous metastatic pancreatic cancer. Participants originated from the multicentre prospective iKnowIT cohort study, which aimed to identify liquid biomarkers for FOLFIRINOX response (Dutch trial register NL75221). All patients received FOLFIRINOX chemotherapy at the Erasmus University Medical Centre Rotterdam (MEC-2018–004), Amsterdam University Medical Centre (L18-053), or Maasstad Hospital (L2018095). FOLFIRINOX chemotherapy was intended to be administered every 2 weeks, and cycles consisted of oxaliplatin (85 mg/m^2^), leucovorin (400 mg/m^2^), irinotecan (180 mg/m^2^), and fluorouracil (400 mg/m^2^ as an intravenous bolus and 2400 mg/m^2^ as a continuous infusion). In accordance with the study protocol, dose modifications were allowed at treatment initiation or during therapy: irinotecan and oxaliplatin could be started at 80% of the standard dose, and the fluorouracil bolus could be omitted in patients with a WHO performance status of 1, age > 75 years, or based on the clinician judgement. In routine practice, treatment intervals were also frequently prolonged, or doses further adjusted in response to adverse events. Chemotherapy was discontinued in case of disease progression, unacceptable toxicity, or at the patient’s request.

### Treatment response evaluation

Treatment response was assessed according to the Response Evaluation Criteria In Solid Tumours (RECIST) 1.1 [12] based on CT or MRI scans. Patients were classified as having either progressive disease or disease control, the latter encompassing complete response, partial response, and stable disease. Imaging assessments were performed at baseline (pre-treatment), after four and eight cycles, after the final cycle (if more than eight cycles were administered), and at 6 months, 1 year, and 2 years post-chemotherapy to evaluate radiologic response and survival over time.

For longitudinal comparison, radiologic outcomes at the completion of FOLFIRINOX were dichotomised: the term ‘response at FOLFIRINOX completion’ refers to disease status at any time point during active treatment, with patients showing progression at any evaluation categorised as having progressive disease, and all others as having disease control. At each follow-up, patients were similarly categorised into progressive disease or disease control, enabling consistent evaluation of treatment response and survival over time. Assessment of chemotherapy-related burden was not included in this study, as it fell outside the original objectives of the iKnowIT study.

### Statistical analyses

Statistical analyses were performed in R Statistical Software (v4.1.2). Clinical and treatment characteristics were compared between patients with LAPC and metastatic PDAC using Fisher’s exact test for categorical variables and the Mann–Whitney *U* test for continuous variables. Overall survival (OS) and progression-free survival (PFS) were estimated from the first FOLFIRINOX cycle to the date of death or last follow-up using the Kaplan–Meier method, with comparisons between groups assessed using the log-rank test. A two-sided *p*-value < 0.05 was considered statistically significant.

## Results

### Clinical characteristics of the advanced PDAC cohort

This study included 84 patients with advanced PDAC, of whom 40 (61%) had LAPC, and 26 (39%) had metastatic PDAC. Amongst them, 18 (21%) patients received only 1–3 cycles of FOLFIRINOX, mainly due to treatment toxicity (*N* = 14) or rapid disease progression (*N* = 4) (Supplementary Table S1), and were therefore excluded from analyses focused on treatment effectiveness after completion of FOLFIRINOX. The baseline characteristics of the 66 patients who received at least four cycles and were included in subsequent analyses are summarised in Table 1. At the completion of FOLFIRINOX treatment, 52 patients (79%) experienced disease control, whilst 14 (21%) experienced disease progression. Treatment response groups were largely comparable with respect to age, sex, BMI, and disease stage at diagnosis. Before the first FOLFIRINOX cycle, CA19-9 levels were significantly higher in patients with progressive disease than in those with disease control at the end of treatment (1700 vs 420 µmol/L; *p* = 0.04), whilst no significant difference was observed before the second cycle (Table 1).

**Table 1:** Clinical characteristics of patients with advanced PDAC who completed ≥ 4 FOLFIRINOX cycles (*N* = 66), stratified by radiologic treatment response at FOLFIRINOX completion^a^

Clinical variable	Group/unit^b^	Disease control at FOLFIRINOX completion^a^ (*N* = 52)	Progressive disease at FOLFIRINOX completion^a^ (*N* = 14)	*p*-value	Total (*N* = 66)
Age at diagnosis	Years	66 (48–82)	62 (49–78)	0.13	65 (48–82)
BMI at diagnosis	Kg/m^2^	25 (16–35)	24 (20–36)	0.81	25 (16–36)
Sex	Female	26 (50%)	6 (43%)	0.77	32 (48%)
	Male	26 (50%)	8 (57%)		34 (52%)
Disease stage at diagnosis	LAPC	34 (65%)	6 (43%)	0.14	40 (61%)
	Metastatic disease	18 (35%)	8 (57%)		26 (39%)
CA19-9 before first FOLFIRINOX cycle	µmol/L	320 (35–26,000)	1800 (550–83,000)	0.04	460 (35–83,000)
CA19-9 before second FOLFIRINOX cycle	µmol/L	420 (33–36,000)	1700 (42–85,000)	0.5	510 (33–85,000)

^a^Patients exhibiting progressive disease at any evaluation time point during active chemotherapy, according to RECIST 1.1, are categorised as having progressive disease; otherwise, they are classified as disease control

^b^Continuous variables are presented as median (range), and categorical variables are presented as frequency (% of total)

### Treatment characteristics

**Fig. 1:**
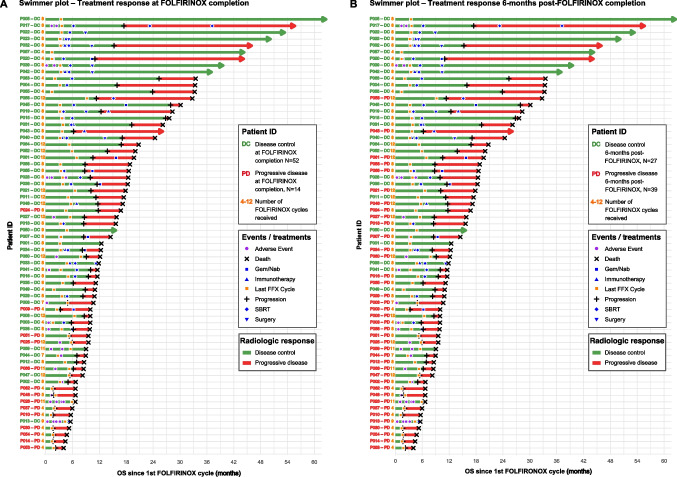
Swimmer plots showing individual patient trajectories and radiologic response in advanced PDAC. Each patient is represented on the *y*-axis with a coloured identifier reflecting (**A**) radiologic response at FOLFIRINOX completion and (**B**) 6 months after FOLFIRINOX completion. Next to each identifier, the yellow number indicates the total number of FOLFIRINOX cycles received. Bars represent treatment timelines, annotated with multiple events including adverse events, progression, death, last FOLFIRINOX cycle, and subsequent therapies

The swimmer plots in Fig. 1 further demonstrate the individual treatment trajectories of patients receiving at least four FOLFIRINOX cycles, including FOLFIRINOX treatment characteristics, therapies after FOLFIRINOX, AEs, and clinical outcomes.

The median FOLFIRINOX treatment duration was 4 months (range 1–9) in first line, and the median number of cycles was 8 (range 4–12) (Table 2). Patients with disease control at FOLFIRINOX completion received significantly more cycles than patients with progressive disease (median of four vs eight cycles; *p* = 0.007). Prophylactic or on-indication G-CSF support was administered to 59 patients (89%), with similar use across treatment response groups (*p* = 0.16). Although not statistically significant (*p* = 0.08), patients with disease control at FOLFIRINOX completion underwent additional therapies after FOLFIRINOX, such as SBRT or surgery, more frequently. Long-term survival was mainly observed in patients who, following disease control during FOLFIRINOX, were eligible for subsequent locoregional treatment, reflecting a selected subgroup. Overall, 36 patients (55%) received subsequent systemic or locoregional therapies, most commonly gemcitabine/nab-paclitaxel (6%), SBRT alone (17%), or immunotherapy combined with SBRT (11%).

**Table 2:** Treatment characteristics of the advanced PDAC cohort (*N* = 66) stratified by radiologic treatment response at FOLFIRINOX completion^a^

Treatment characteristic	Group/unit^b^	Disease control at FOLFIRINOX completion^a^ (*N* = 52)	Progressive disease at FOLFIRINOX completion^a^ (*N* = 14)	*p*-value	Total (*N* = 66)
G-CSF received	No	4 (8%)	3 (21%)	0.16	7 (11%)
	Yes	48 (92%)	11 (79%)		59 (89%)
Number of FOLFIRINOX cycles received		8 (4–12)	4 (4–12)	0.007	8 (4–12)
Number of FOLFIRINOX-related grade ≥ 3 AEs	No AE	35 (67%)	9 (64%)	0.38	44 (67%)
	1 AE	9 (17%)	2 (14%)		11 (17%)
	2 AEs	3 (6%)	2 (14%)		5 (8%)
	3 AEs	2 (4%)	0 (0%)		2 (3%)
	4 AEs	3 (6%)	0 (0%)		3 (5%)
	5 AEs	0 (0%)	1 (7%)		1 (2%)
Additional therapies after FOLFIRINOX	No other therapy	17 (33%)	13 (93%)	0.08	30 (45%)
	Gem/nab	3 (6%)	1 (7%)		4 (6%)
	Immunotherapy	2 (4%)	0 (0%)		2 (3%)
	Immunotherapy + SBRT	1 (2%)	0 (0%)		1 (2%)
	Immunotherapy + SBRT + Gem/nab	2 (4%)	0 (0%)		2 (3%)
	Immunotherapy + SBRT + Surgery	6 (12%)	0 (0%)		6 (9%)
	Other (IRE, targeted)	4 (8%)	0 (0%)		4 (6%)
	SBRT	11 (21%)	0 (0%)		11 (17%)
	SBRT + Gem/nab	3 (6%)	0 (0%)		3 (5%)
	SBRT + Surgery	2 (4%)	0 (0%)		2 (3%)
	Surgery	1 (2%)	0 (0%)		1 (2%)

^a^Patients exhibiting progressive disease at any evaluation time point during active chemotherapy, according to RECIST 1.1, are categorised as having progressive disease; otherwise, they are classified as disease control

^b^Continuous variables are presented as median (range), and categorical variables are presented as frequency (% of total)

### Adverse events

Of the original 84 patients, 14 (17%) experienced grade ≥ 3 toxicities that prevented completion of four FOLFIRINOX cycles. Amongst the remaining 66 patients who completed at least four cycles, FOLFIRINOX-related AEs of grade ≥ 3 occurred in 22 patients (33%), with a single grade ≥ 3 AE in 17%, two events in 8%, three in 3%, and four or more in 7% of patients. One treatment-related death occurred due to bacterial sepsis.

The overall incidence of grade ≥ 3 AEs did not differ significantly between patients achieving disease control and those with progressive disease at FOLFIRINOX completion (33% vs 36%; *p* = 0.38) (Table 2). Similarly, AE frequency was not associated with disease stage, sex, or the number of FOLFIRINOX cycles administered (Supplementary Table S2).

Amongst specific grade ≥ 3 events, anaemia, diarrhoea, and infection were most frequent (each > 10% of all severe AEs), whereas hypokalaemia, nausea, and pulmonary embolism were less common. The frequency and types of grade ≥ 3 AEs were similar across subgroups based on radiologic response at different time points (Supplementary Tables S3 and S4).

### Radiologic responses during and after FOLFIRINOX

Radiologic response was evaluated at sequential time points during and after FOLFIRINOX (Table 3). At the first radiologic evaluation after four cycles, 55 of 66 patients (83%) demonstrated disease control, whilst 11 (17%) had progressive disease. Amongst the 52 patients who continued past four cycles, 48 (92%) maintained disease control through cycles 5–8. Similarly, of the 17 patients treated beyond eight cycles, 14 (82%) maintained disease control through cycles 9–12.

**Table 3:** Treatment responses and survival outcomes at sequential time points during and after FOLFIRINOX treatment

Time points for radiologic response evaluation	Response to FOLFIRINOX	Total, *N*^a^	Median OS since first cycle FOLFIRINOX in months^a^	Log-rank *p*-value^e^	Median PFS since first cycle FOLFIRINOX in months^a^	Log-rank *p*-value^e^
Response during chemotherapy after 4 cycles	Disease control	55 (83%)	17.8 (5.6–61.6)	< 0.001	10.5 (4.9–61.6)	< 0.001
	Progressive disease	11 (17%)	5.9 (4.0–16.8)		1.8 (1.6–11.7)	
Response during chemotherapy after 5–8 cycles^c^	Disease control	48 (92%)	17.4 (5.6–61.6)	0.009	10.1 (4.9–61.6)	0.014
	Progressive disease	4 (8%)	9.6 (6.7–16.8)		5.6 (1.8–11.7)	
Response during chemotherapy after 9–12 cycles^d^	Disease control	14 (82%)	17.2 (5.6–32.8)	0.008	10.5 (5.4–16.9)	0.021
	Progressive disease	3 (18%)	8.5 (4.0–9.6)		6.2 (6.0–6.2)	
Response at FOLFIRINOX completion^b^	Disease control	52 (79%)	18.1 (5.6–61.6)	< 0.001	10.8 (4.9–61.6)	< 0.001
	Progressive disease	14 (21%)	6.7 (4.0–16.8)		2.2 (1.6–11.7)	
Response 6-months post-FOLFIRINOX completion	Disease control	27 (41%)	28.3 (11.0–61.6)	< 0.001	16.9 (8.8–61.6)	< 0.001
	Progressive disease	19 (29%)	15.7 (9.0–32.8)		8.5 (3.3–11.7)	
	Diseased due to progression	20 (30%)	6.7 (4.0–12.2)		5.1 (1.6–9.1)	
Response 1-year post-FOLFIRINOX completion	Disease control	14 (21%)	34.8 (24.4–61.6)	< 0.001	27.3 (16.0–61.6)	< 0.001
	Progressive disease	15 (23%)	19.7 (15.7–45.0)		11.2 (6.2–16.9)	
	Diseased due to progression	37 (56%)	9.7 (4.0–17.8)		6.2 (1.6–14.7)	
Response 2-years post-FOLFIRINOX completion	Disease control	7 (11%)	43.3 (30.1–61.6)	< 0.001	43.3 (27.9–61.6)	< 0.001
	Progressive disease	7 (11%)	33.5 (28.3–54.7)		16.0 (11.0–25.3)	
	Diseased due to progression	52 (79%)	11.4 (4.0–32.8)		8.5 (1.6–26.8)	

^a^Continuous variables are presented as median (range), and categorical variables are presented as frequency (% of total)

^b^Patients were classified as having progressive disease if progression was observed at any evaluation time point during active chemotherapy according to RECIST 1.1; all others were categorised as disease control

^c^A total of 14 (21%) patients are missing because these patients received < 5 cycles of FOLFIRINOX

^d^A total of 49 (74%) patients are missing because these patients received < 9 cycles of FOLFIRINOX

^e^Log-rank *p*-value was calculated between patients with disease control and those with progressive disease. If ‘diseased due to progression’ existed, this group was combined with the ‘progressive disease’ group

As patients received varying cycles of FOLFIRINOX, radiologic outcomes at FOLFIRINOX completion were dichotomised for accurate longitudinal analyses. Amongst 66 patients treated with FOLFIRINOX, 52 (79%) had disease control at the evaluation performed at the time of FOLFIRINOX completion, whereas 14 (21%) experienced progressive disease at that time point. However, this treatment response was not durable. Six months post-FOLFIRINOX completion, disease control persisted in 27 patients (41%), whereas 19 (29%) had progressed, and 20 (30%) had died. One year post-FOLFIRINOX completion, the disease control rate further declined to 14 patients (21%), whilst 15 patients (23%) showed progression, and 37 patients died (56%). By 2 years post-FOLFIRINOX completion, disease control rates continued to decline to seven patients (11%), whilst seven patients (11%) showed progression, and 52 died (79%) (Table 3). Swimmer plots further illustrate that initial disease control during FOLFIRINOX or at FOLFIRINOX completion often progressed to death, with patient identifiers labelled by radiologic outcome during treatment (Fig. 1A) and by status 6 months later (Fig. 1B). It was unlikely that prolonged disease control was attributable to subsequent adjuvant treatment, as patients with disease control did not receive adjuvant therapies more frequently than those who progressed or died (Supplementary Table S1).

### Survival outcomes according to radiologic responses to FOLFIRINOX

**Fig. 2:**
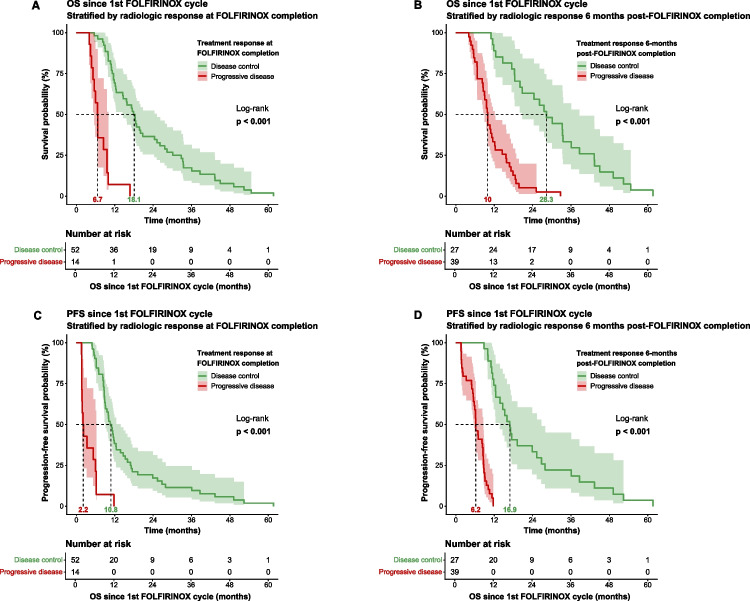
Kaplan–Meier curves for OS and PFS stratified by radiologic response. OS (**A**) and PFS (**C**) according to treatment response at FOLFIRINOX completion. OS (**B**) and PFS (**D**) according to treatment response 6 months after completing FOLFIRINOX treatment. Median survival times are shown in the figure, and the log-rank test was used to assess statistical significance

OS and PFS were evaluated according to radiologic response at sequential time points during and after FOLFIRINOX (Table 3). Patients with disease control at their individual time of FOLFIRINOX completion (52 of 66, 79%) had significantly longer survival, calculated from the first FOLFIRINOX cycle, compared with patients who experienced progressive disease (median OS 18.1 vs 6.7 months; median PFS 10.8 vs 2.2 months; *p* < 0.001) (Fig. 2A, C). Prolonged survival was observed predominantly in patients eligible for subsequent locoregional therapy. Six months after completing therapy, fewer patients maintained disease control (27 of 66, 41%), and sustained disease control remained associated with improved outcomes (median OS 28.3 vs 10.0 months; *p* < 0.001) (Fig. 2B, D). At 1 and 2 years after FOLFIRINOX, a minority of patients maintained disease control (14 of 66, 21% at 1 year; 7 of 66, 11% at 2 years) (Table 3), and those who did, continued to experience prolonged survival (Supplementary Fig. S1).

### Impact of clinical and treatment characteristics on radiologic response

Stratified analyses were performed to investigate the influence of clinical characteristics and the number of FOLFIRINOX cycles on radiologic response and survival. Disease stage was a determinant of radiologic outcomes, with LAPC patients more likely than metastatic patients to achieve disease control 6 months (*p* < 0.001), 1 year (*p* < 0.002), and 2 years (*p* < 0.01) post-FOLFIRINOX completion. Nonetheless, patients of both disease stages showed a decreasing pattern in disease control (Supplementary Table S5). In patients with LAPC, disease control dropped from 85% at FOLFIRINOX completion to 55% after 6 months, 32% after 1 year, and 15% after 2 years. In patients with metastatic PDAC, disease control declined from 69% at FOLFIRINOX completion to 19% after 6 months and 4% after 1 year and 2 years. Sex was not associated with radiologic treatment response (Supplementary Table S5).

Patients receiving 5–8 FOLFIRINOX cycles appeared to maintain disease control more often than those receiving 9–12 cycles at 6 months (*p* < 0.04) and 1 year (*p* < 0.03) post-FOLFIRINOX completion (Supplementary Table S5). However, this difference was likely confounded by an imbalance in disease stage between the groups. The 9–12 cycle group included predominantly patients with metastatic disease (15 of 17, 88%), whilst the 5–8 cycle group included mostly LAPC patients (28 of 35, 80%) (Supplementary Table S6 and Fig. S2). Small subgroup sizes prevented meaningful stratified comparisons between cycle-number groups within each disease stage. Consistent with radiologic findings, OS and PFS were primarily determined by disease stage rather than the number of FOLFIRINOX cycles (Supplementary Fig. S3). LAPC patients had longer median OS (18.5 vs 10.3 months; *p* = 0.006) and PFS (11.2 vs 7.8 months; *p* = 0.04) than metastatic patients.

## Discussion

Defining what constitutes valuable FOLFIRINOX chemotherapy is becoming increasingly important as clinicians strive to balance efficacy, toxicity, cost, and patient well-being. Importantly, growing evidence suggests that gemcitabine-based combination regimens can achieve survival outcomes comparable to FOLFIRINOX in certain cases of advanced disease and in some neoadjuvant scenarios. This shift directs clinical focus from simply comparing regimens to prioritising patient selection, early benefit reassessment, and the durability of disease control when opting for intensive chemotherapy. This study contributes to that discussion by demonstrating that, although FOLFIRINOX can induce short-term tumour control, these effects often decline rapidly after treatment completion. Relying solely on radiologic or biochemical responses provides an incomplete measure of therapeutic value. Instead, value should be assessed through a multidimensional lens that includes durability of response, toxicity burden, QoL, and wider ethical and financial considerations that inform further shared decision-making.

### High toxicity limits treatment continuation

Consistent with existing literature, FOLFIRINOX was highly toxic in our real-world cohort, restricting treatment continuation [5, 13–15]. One in five patients could not complete four cycles due to grade ≥ 3 adverse events. Following four cycles, almost one in ten patients discontinued FOLFIRINOX despite disease control due to toxicity. In total, one in four patients could not receive more than four cycles of FOLFIRINOX because of severe toxicity, and more than four out of ten experienced at least one grade ≥ 3 FOLFIRINOX-related AE during treatment.

### Initial disease control is high, but durability is poor

Despite the toxicity burden, initial radiologic response rates were high in our cohort. After four cycles, 83% of evaluable patients achieved disease control, and amongst those able to continue treatment, disease control rates remained favourable (92% after 5–8 cycles and 82% after 9–12 cycles). Initial disease control rates in our cohort were slightly higher than those reported in recent prospective and real-world studies, which describe disease control rates of approximately 77–82% after four and eight cycles of FOLFIRINOX. This difference likely reflects the selection of patients able to continue treatment beyond early cycles [5, 10, 15–17]. However, these benefits proved to be short-lived: disease control rates dropped to 41% at 6 months, 21% at 1 year, and 11% at 2 years after FOLFIRINOX completion. Consistent with this observation, published studies report a rapid decline in disease control following completion of FOLFIRINOX, with rates decreasing to approximately 41% at 6 months, 21% at 1 year, and 11% at 2 years [5, 10, 15]. Many patients who initially responded subsequently progressed or died within months, suggesting that disease control during or at completion of FOLFIRINOX does not reliably translate into sustained benefit.

We also examined whether clinical characteristics or the number of FOLFIRINOX cycles influenced radiologic or survival outcomes. Disease stage was a major determinant, with LAPC patients achieving disease control and prolonged survival more frequently than those with metastatic disease. This finding is consistent with prior cohort studies and meta-analyses showing substantially longer survival in locally advanced compared with metastatic disease when treated with FOLFIRINOX-based regimens [5, 13–15]. Nevertheless, both groups experienced steep declines in disease control over time, from 85 and 69% at FOLFIRINOX completion to 32% and 4% 1 year in LAPC and metastatic PDAC, respectively. Similar post-treatment attrition in disease control has been reported in the literature, underscoring the aggressive biology of pancreatic cancer and the limited durability of response irrespective of initial disease stage [5, 13, 15, 18].

Apparent differences between cycle-number groups (e.g. seemingly better outcomes in the 5–8 cycle group compared with the 9–12 cycle group) were largely explained by imbalances in disease stage. Unfortunately, small subgroup sizes hindered stage-stratified comparisons, preventing meaningful conclusions about the benefits of specific cycle durations. Larger, prospective studies will be required to address the impact of cycle number on treatment durability more definitively.

### Clinical meaning of the 6-month post-treatment milestone

The 6-month post-treatment time point was clinically meaningful: patients who maintained disease control beyond this time point experienced substantially longer OS and PFS. These findings align with existing literature. In LAPC, median PFS ranges from 3 to 20 months, typically around 15 months [5], whereas in metastatic PDAC, median PFS is typically 5.6–6.0 months [6, 7, 19, 20]. Conroy et al. reported a 6-month PFS of 52.8% in metastatic PDAC, higher than the 40% observed in our mixed cohort [8]. Together with our results, these data reinforce that although FOLFIRINOX can induce early tumour shrinkage, its long-term efficacy in advanced PDAC is limited, underscoring the need for close post-treatment monitoring and more durable therapeutic strategies.

### Limitations in current response evaluation

In clinical practice, the response to chemotherapy is primarily determined by RECIST 1.1 imaging. Yet radiological assessments have inherent limitations. Tumour size reduction often lags biological activity, meaning that early responses can go undetected. Moreover, distinguishing true progression from pseudo-progression remains challenging, as peri-tumoral inflammation may transiently increase lesion size. This ambiguity contributes to high inter-observer variability amongst radiologists [21, 22]. Small metastatic lesions may also be overlooked, and several studies have demonstrated the poor predictive value of contrast-enhanced CT or MRI in assessing response to FOLFIRINOX in advanced pancreatic cancer [21–23]. In addition, the financial and logistical burden of repeated imaging adds to the overall challenges of routine monitoring. Together, these limitations underscore the need for multidimensional and multimodal assessment strategies.

Circulating biomarkers, particularly CA19-9, offer a complementary and less invasive means of real-time monitoring of treatment response [7, 24, 25]. Longitudinal CA19-9 measurements identify progression earlier than imaging and help refine treatment timing. In the future, data-driven predictive models based on artificial intelligence could integrate biomarkers, demographics, and clinical parameters to forecast recurrence or mortality risk more accurately.

Nonetheless, biomarkers like CA19-9 have constraints. CA19-9 is not a reliable early predictor of FOLFIRINOX response, only becoming indicative after multiple cycles [7, 24–27]. Moreover, 10–20% of individuals do not express CA19-9 at all, and inter- and intra-person variability in biomarker levels complicates the establishment of a universal threshold for defining response [7, 24, 25].

### Early prediction of FOLFIRINOX benefit to avoid overtreatment

These limitations in routine response assessment underscore an unmet clinical need: identifying, as early as possible, which patients are unlikely to derive meaningful benefit from FOLFIRINOX. In translational studies from our group, a single cycle of FOLFIRINOX was shown to induce measurable changes in the peripheral immune landscape and plasma proteome, with baseline VEGFA and PRDX3 levels and early increases in FCRL3 associated with early disease progression. In parallel, an eight-gene immune transcriptomic score (FFX-ΔGEP) assessed after one cycle showed good discriminatory performance in predicting lack of response in exploratory analyses. Whilst promising, these approaches require prospective validation before clinical implementation [26, 28, 29]. Parallel developments in circulating biomarkers further highlight the potential for earlier response assessment during FOLFIRINOX. CA19-9 remains the most widely used biomarker in clinical practice, with early declines after one or two cycles correlating with improved survival and radiologic response. However, its utility for early prediction of non-response is limited by false-negative results in Lewis antigen-negative patients, confounded by biliary obstruction, and substantial interindividual variability; integration with radiologic assessment improves prognostic stratification, but CA19-9 alone may be insufficient for early decision-making [25, 30].

Liquid biopsy approaches offer complementary insights. Early changes in circulating mutant KRAS DNA after a single chemotherapy cycle have been associated with treatment response and survival. However, baseline KRAS status has limited prognostic value, and longitudinal monitoring remains investigational [31–33]. Similarly, recent studies indicate that clearance of TP53 mutations from ctDNA after one cycle is associated with radiologic response, even in settings where CA19-9 is unreliable, suggesting that dynamic ctDNA profiling may provide greater sensitivity for early response assessment [32–34]. Despite these advances, none of these biomarkers are currently guideline-endorsed for routine clinical decision-making, and broader validation and standardisation in independent cohorts are required before clinical implementation. Clinically, however, the overarching goal is clear: earlier identification of patients unlikely to benefit could enable timely treatment adaptation or discontinuation, reduce avoidable toxicity, and support more patient-centred, value-based care.

### Quality of life and person-centred communication

QoL is a cornerstone of oncology care, encompassing physical, psychological, and social well-being [35]. Its meaning varies by patient and is influenced by socioeconomic background, cultural context, medical history, treatment intent, and disease stage [36–38]. Although standardised QoL questionnaires may provide useful data [39], they often fail to capture the personal dimensions of living with PDAC. Prior studies in PDAC patients have shown that palliative care interventions can improve QoL [40]. Similarly, Gehrels et al*.* observed that in patients with metastatic pancreatic cancer, chemotherapy, including FOLFIRINOX, can alleviate disease-related symptoms and thereby help maintain QoL, although treatment-related toxicities may partially offset these benefits [41]. Yet the magnitude of QoL improvement varies between individuals. For example, van der Sijdeet al*.* observed that patients with LAPC who achieved disease control following FOLFIRINOX reported superior QoL scores compared to cancer patients and the general population norms [42], whereas patients with progressive disease typically experience marked deterioration across all QoL domains [43, 44]. This parallel between radiologic response and patient-reported outcomes reinforces that sustained disease control not only prolongs survival but also preserves QoL.

Determining whether a treatment is ‘bearable’ or ‘worthwhile’ is inherently subjective [45] and depends on coping capacity [46] and individual trade-offs between QoL and longevity [47, 48]. Such perceptions also differ according to therapeutic intent, curative or palliative [49]. In the context of advanced PDAC, transparent communication about prognosis and toxicity is essential. Patients must understand that the objective is not to cure, but to extend life. In our study, one in three patients discontinued FOLFIRINOX due to toxicity, with one treatment-related death. Such figures highlight the ethical tension between pursuing aggressive treatment and preserving QoL.

Effective, personalised communication is therefore essential, focusing not only on prognosis but also on the burden of treatment. Reports showed that for discussions to be productive and meaningful, they should be tailored to the patient’s level of understanding, values, and emotional needs [50, 51]. Also, evidence suggests that cancer patients who better understand their condition report higher QoL [52]. Moreover, involving family and caregivers enhances coping, mitigates distress, and improves both patient and caregiver well-being [37, 51, 53, 54]. Although these discussions can be time-consuming, their long-term benefits are substantial, including reduced hospital admissions, improved adherence, and more realistic expectations. Innovative frameworks, such as ‘Metro Mapping’, may offer structured, service-design-based approaches to support shared decision-making for patients, their significant others, and healthcare professionals whilst overcoming the problem of being time-consuming [51, 55]. In addition, early management of side effects and the integration of supportive interventions, such as physical exercise and nutritional optimisation, can improve QoL and decrease end-of-life hospitalisations [51, 53, 56].

Nevertheless, implementing person-centred care remains challenging. Clinicians often face barriers such as limited time, discomfort discussing mortality, and variability in perceptions of what constitutes valuable therapy [50]. Still, incorporating routine QoL assessments and goal-setting discussions into treatment plans is essential for aligning medical care with patient priorities [36, 51, 55].

### Financial considerations

The financial dimension of chemotherapy is inseparable from its clinical and ethical evaluation. When a treatment as intensive as FOLFIRINOX provides only temporary disease control and has high toxicity, its cost-effectiveness needs evaluation. Chemotherapy-related complications are not only burdensome to patients but can also elevate healthcare expenses. Therefore, identifying appropriate candidates for FOLFIRINOX early is not only clinically but also economically important.

Although financial factors should never dictate individual treatment decisions, they are central to healthcare sustainability. Cost-effectiveness, often expressed in quality-adjusted life years (QALYs), indicates that FOLFIRINOX is generally more cost-effective than gemcitabine-based regimens in selected settings [57–59]. In the neoadjuvant setting, FOLFIRINOX has shown greater cost-effectiveness for borderline resectable and LAPC compared with gemcitabine-based regimens [15, 60, 61]. In the palliative setting, FOLFIRINOX was found to be more cost-effective than gemcitabine monotherapy [62], but less so than gemcitabine/nab-paclitaxel in certain metastatic contexts [63–66]. Real-world cost analyses further suggest that FOLFIRINOX is associated with lower mean post-treatment costs and fewer hospitalisations compared with gemcitabine plus nab-paclitaxel, despite higher upfront toxicity, whereas it remains more expensive than gemcitabine monotherapy [59, 67]. Importantly, cost-effectiveness varies substantially by disease stage, treatment intent, and durability of response. Therefore, the value of FOLFIRINOX should be contextualised within the expected clinical benefit, reinforcing the need for strategies that enable earlier treatment selection and avoidance of ineffective therapy.

### Study limitations and strengths

This study has limitations. Although patients were drawn from the prospective iKnowIT trial, short-term follow-up data were collected retrospectively. Consequently, the sample size has limited statistical power and precluded detailed subgroup analyses. In addition, locally advanced and metastatic PDAC were analysed within a single cohort, despite important differences in treatment intent and tumour biology, meaning that radiologic disease control may carry different clinical implications across disease stages. Treatment heterogeneity, for example, differences in adjuvant treatments, may have influenced long-term survival outcomes. Furthermore, QoL data were unavailable, preventing correlation with survival and toxicity outcomes. Future research should incorporate QoL measurements alongside clinical and economic data to achieve a more comprehensive assessment of treatment value.

Despite these constraints, the study’s strengths are considerable. It draws on data from three high-volume Dutch hospitals, lending external validity and clinical relevance. The inclusion of toxicity data provides objective insight into treatment burden, complementing the absence of patient-reported outcomes. Most importantly, by capturing both intra-treatment and post-treatment dynamics, this study demonstrates how transient FOLFIRINOX benefits can be and underscores the importance of evaluating short-term follow-up in defining ‘valuable FOLFIRINOX chemotherapy’.

## Conclusion

This study demonstrates that the conventional definition of ‘valuable FOLFIRINOX chemotherapy’, based solely on radiological and biochemical responses during FOLFIRINOX or at completion, fails to capture the broader clinical and ethical picture in patients with advanced PDAC. The high burden of serious AEs and the rapid decline in disease control following treatment completion highlight the limitations of short-term response metrics. A redefinition is needed: one that integrates durability of disease control, toxicity burden, QoL, individual patient goals, and cost considerations.

Engaging patients and their families in open, transparent dialogue is fundamental to aligning treatment with personal values and expectations. Integrating predictive biomarkers, AI-based analytical tools, and structured shared decision-making frameworks may support a transition towards more precise, compassionate, and person-centred cancer care. Ultimately, the real value of FOLFIRINOX chemotherapy lies not only in extending survival, but also in ensuring that this extended life has meaning and quality for those who live it.

## Supplementary Information

Below is the link to the electronic supplementary material.

ESM 1 (PDF 93.1 KB)

ESM 2 (XLSX 28.9 KB)

## Data Availability

The datasets used during the current study are available from the corresponding author upon reasonable request.
