# Supplementary figures and images for: The value of FOLFIRINOX in advanced pancreatic cancer: balancing efficacy, toxicity, and quality of life
Source: Support Care Cancer. 2026 Mar 26;34(4):372. doi: 10.1007/s00520-026-10568-3 (PMC13021859; doi:10.1007/s00520-026-10568-3)

Figure S1

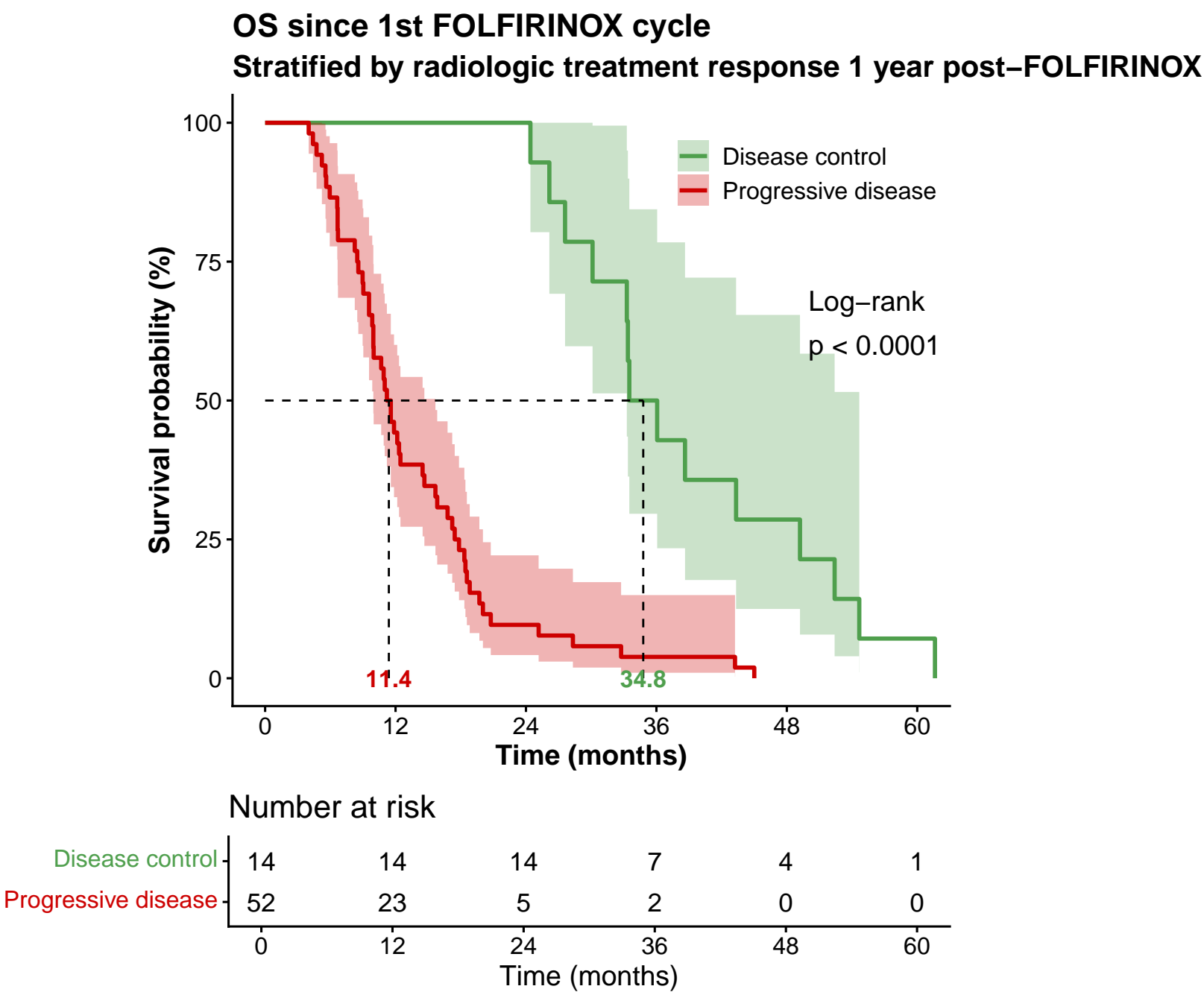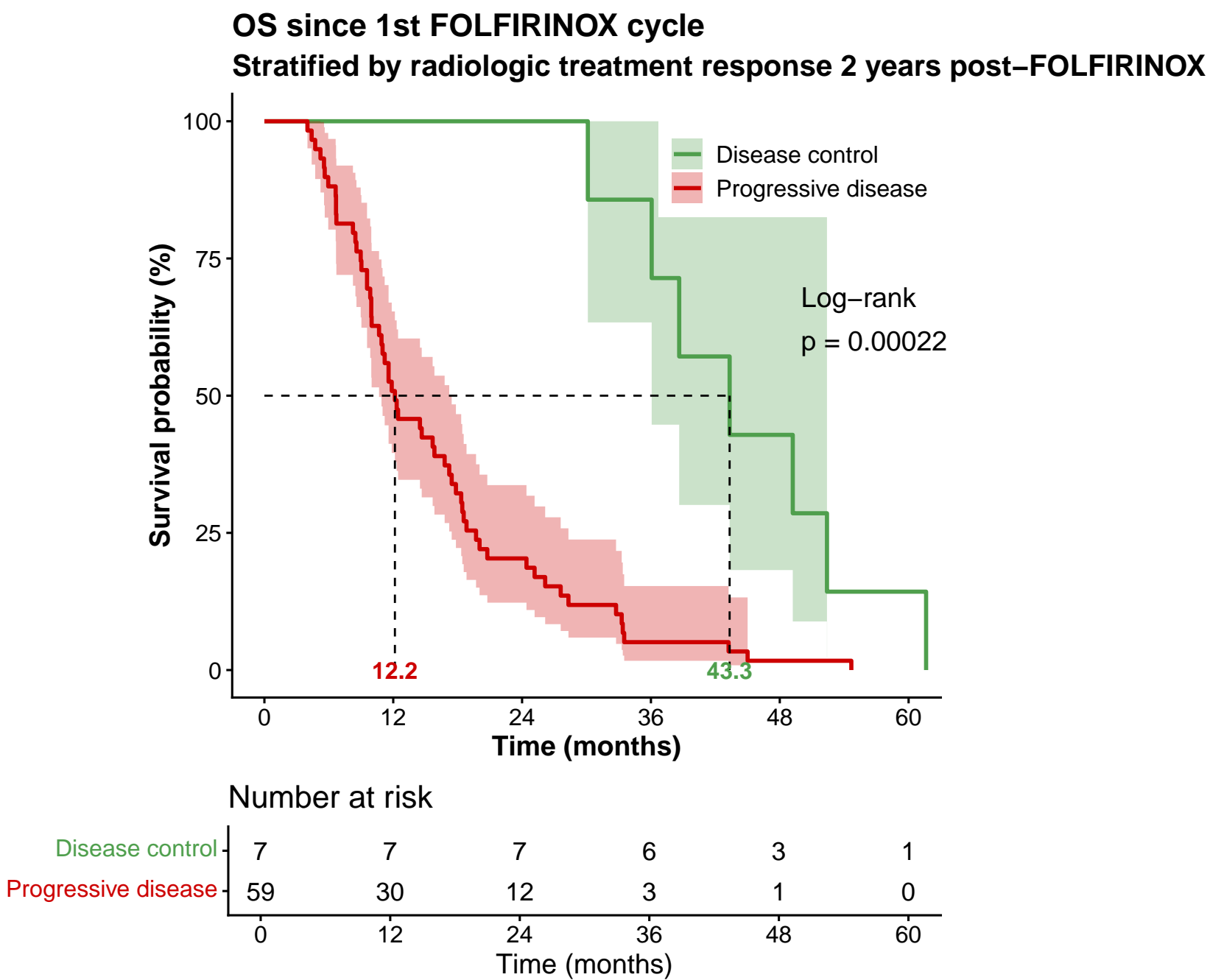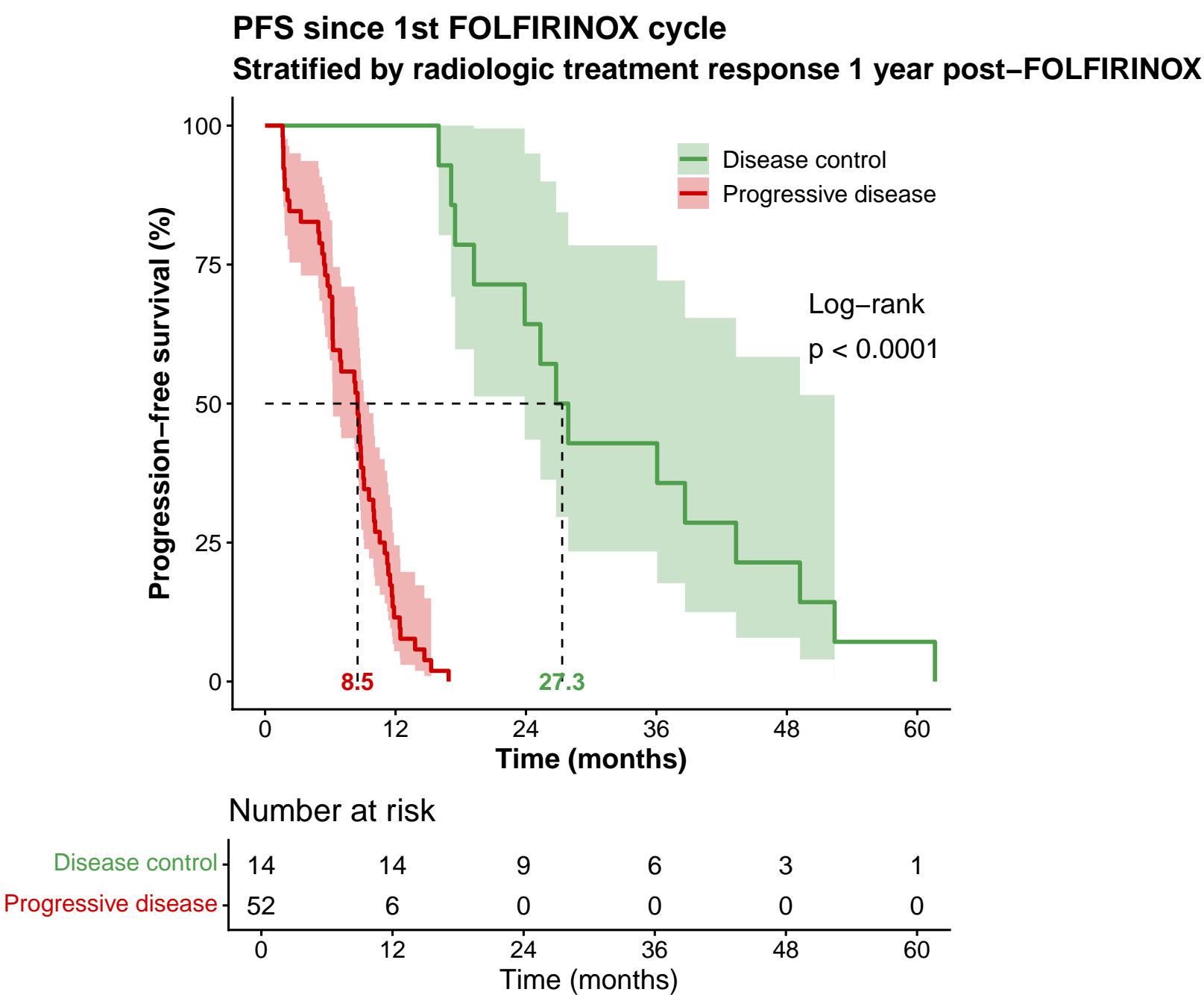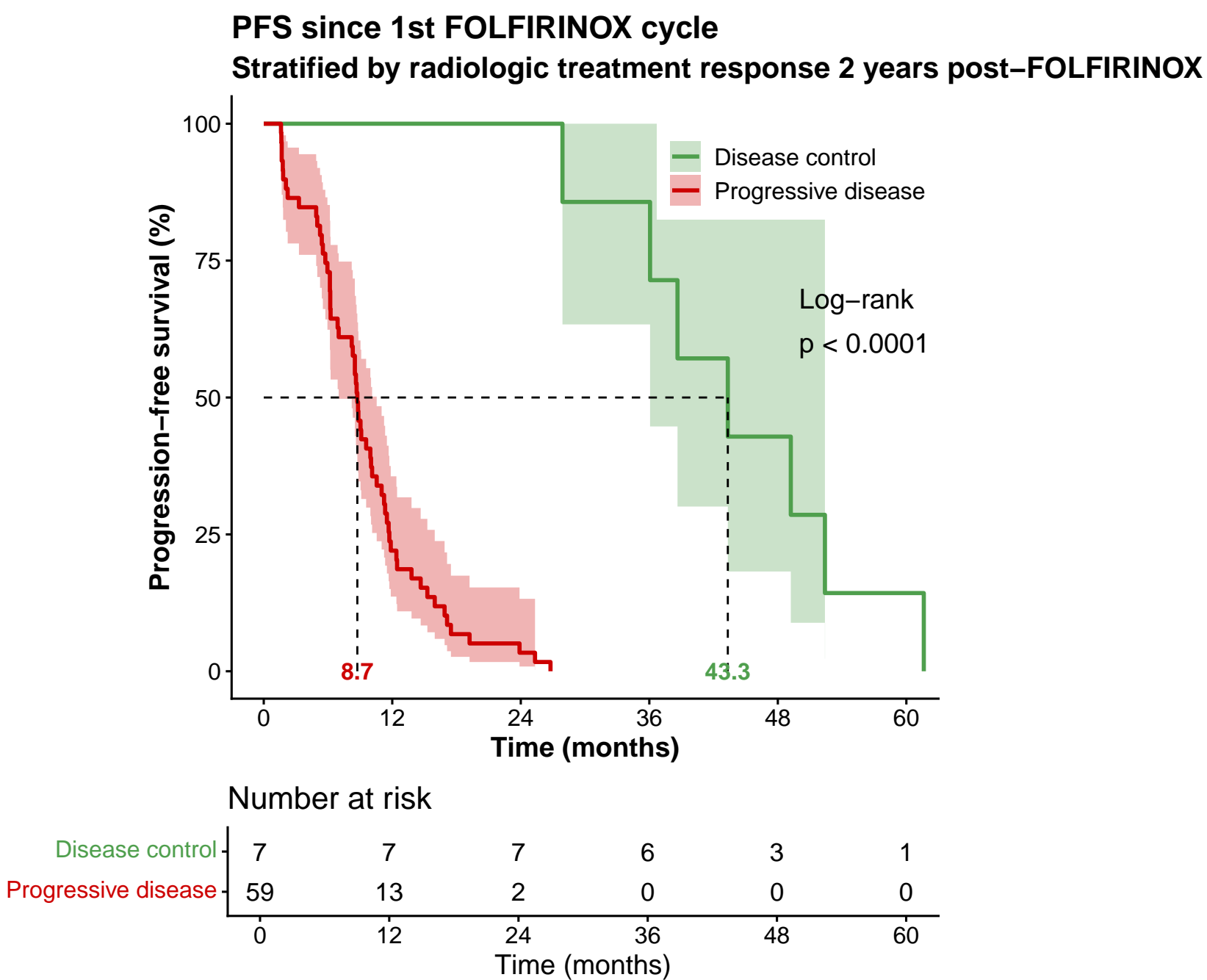

Figure S2

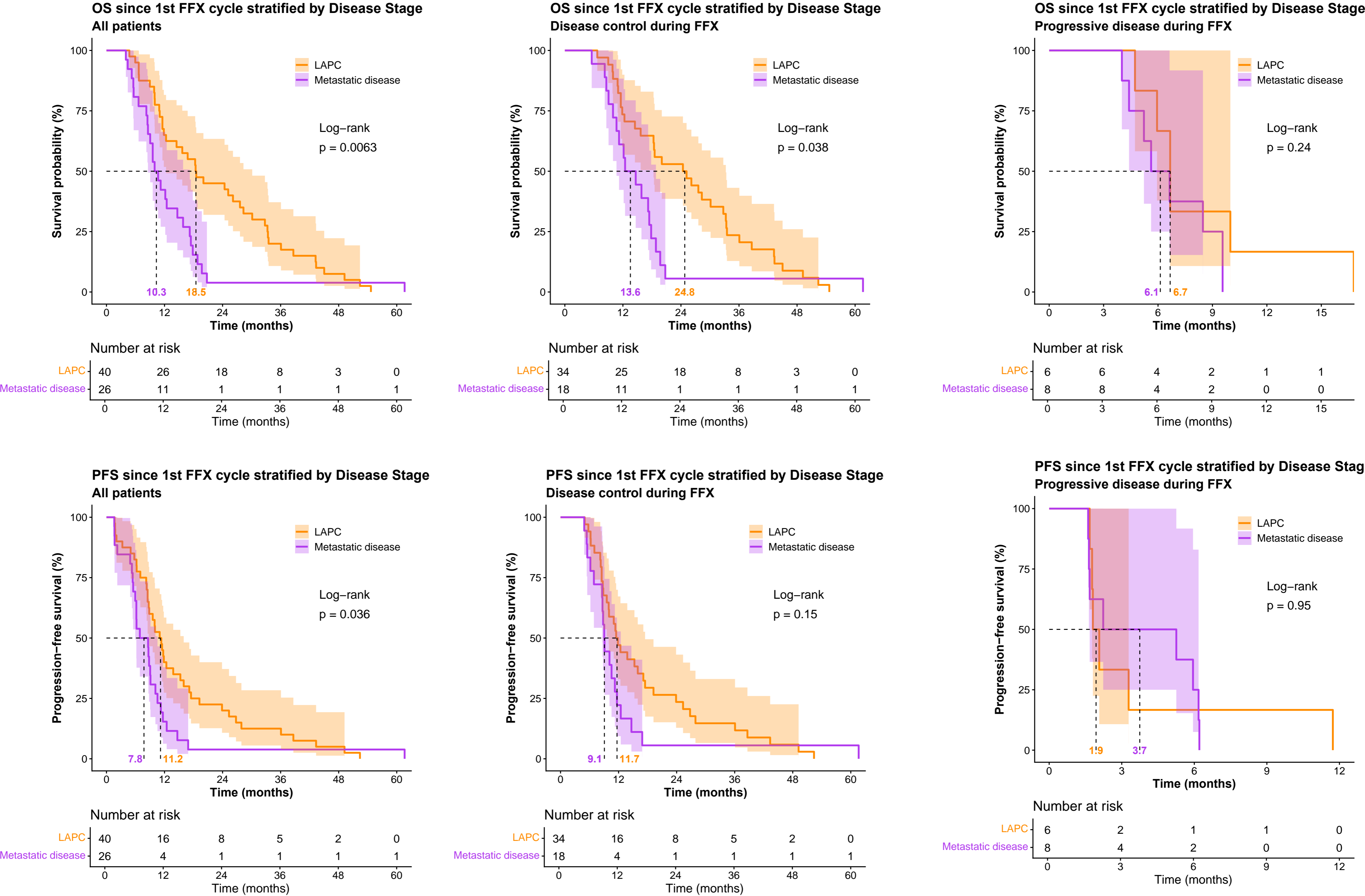

Figure S3

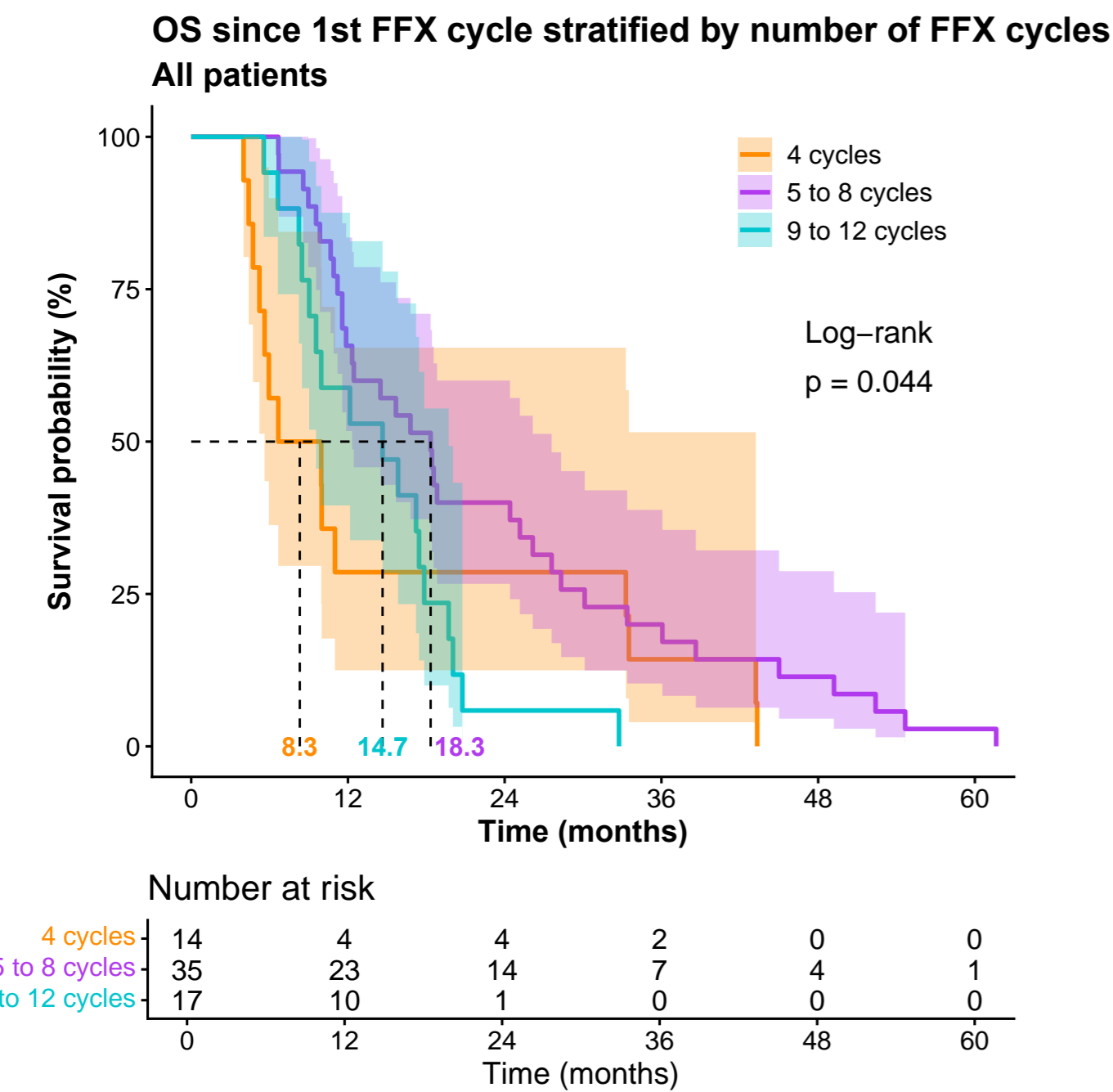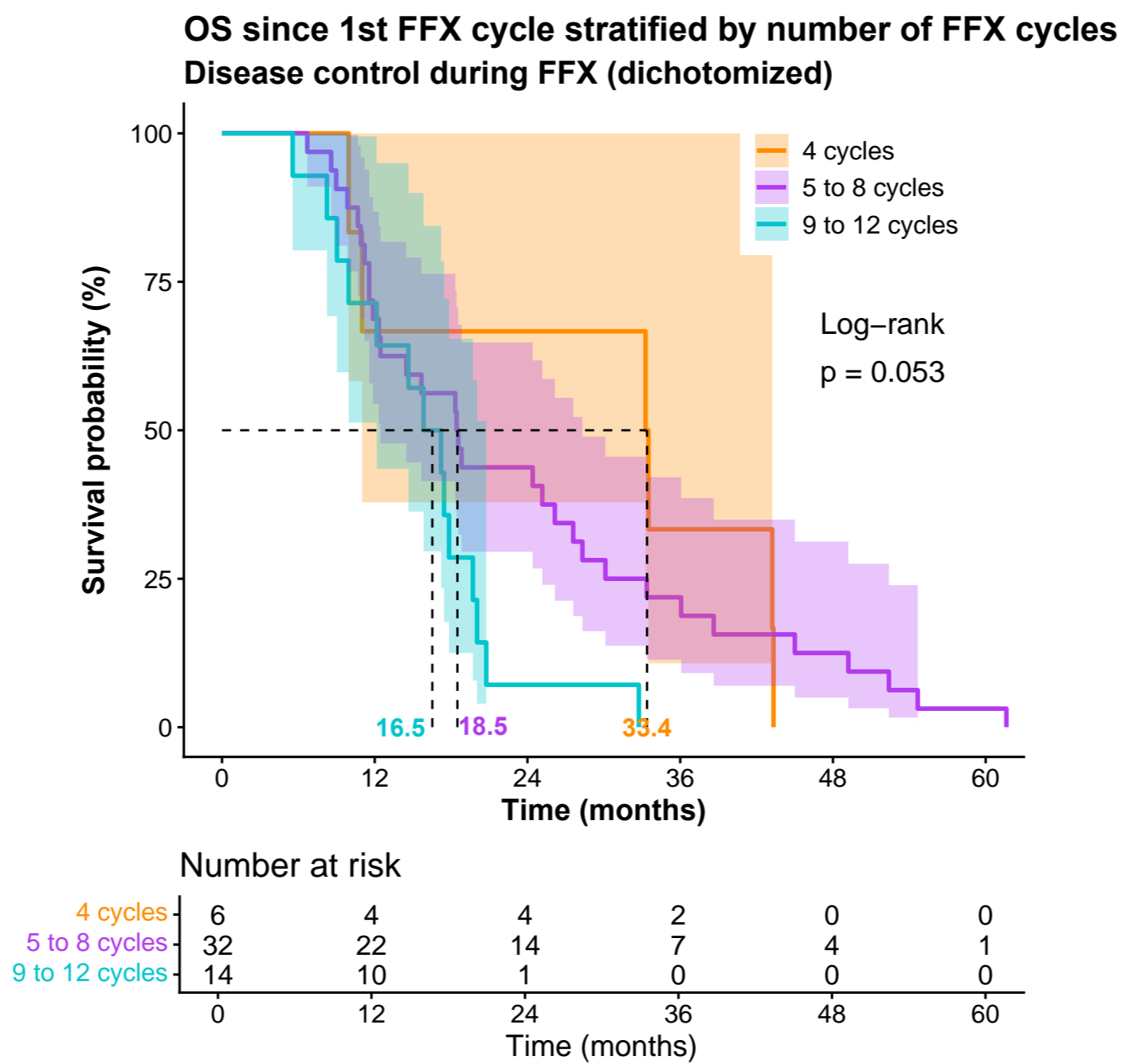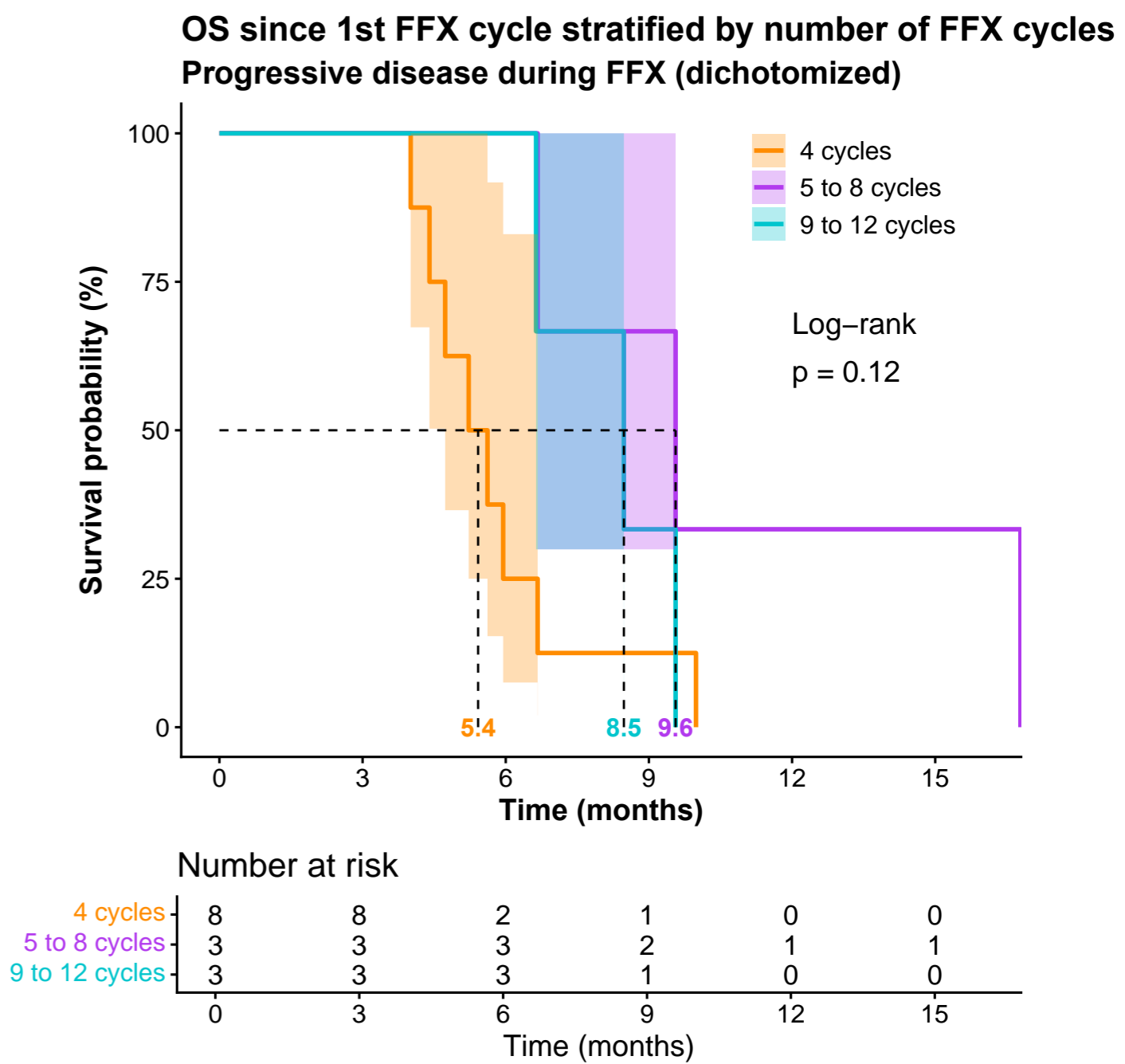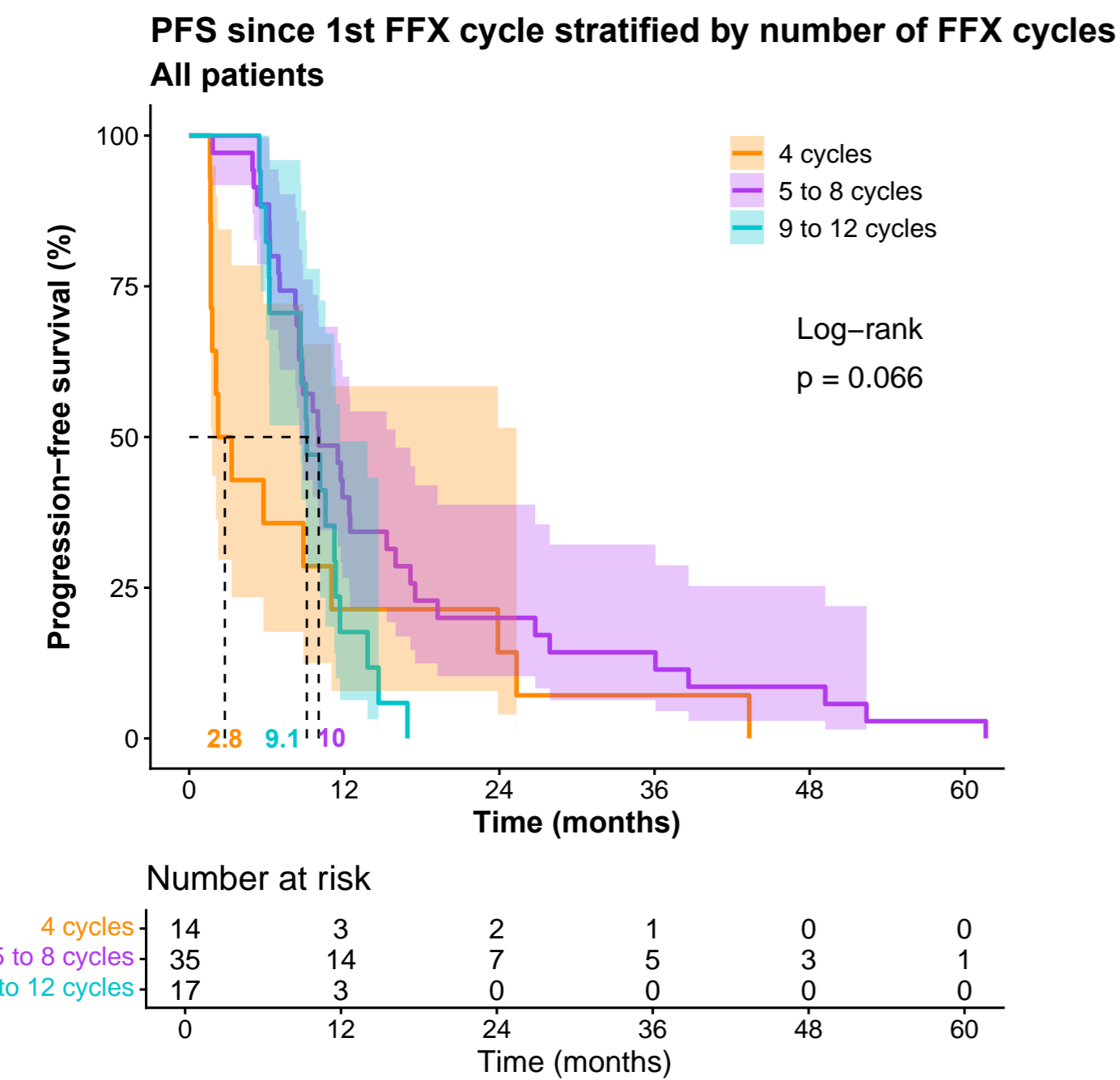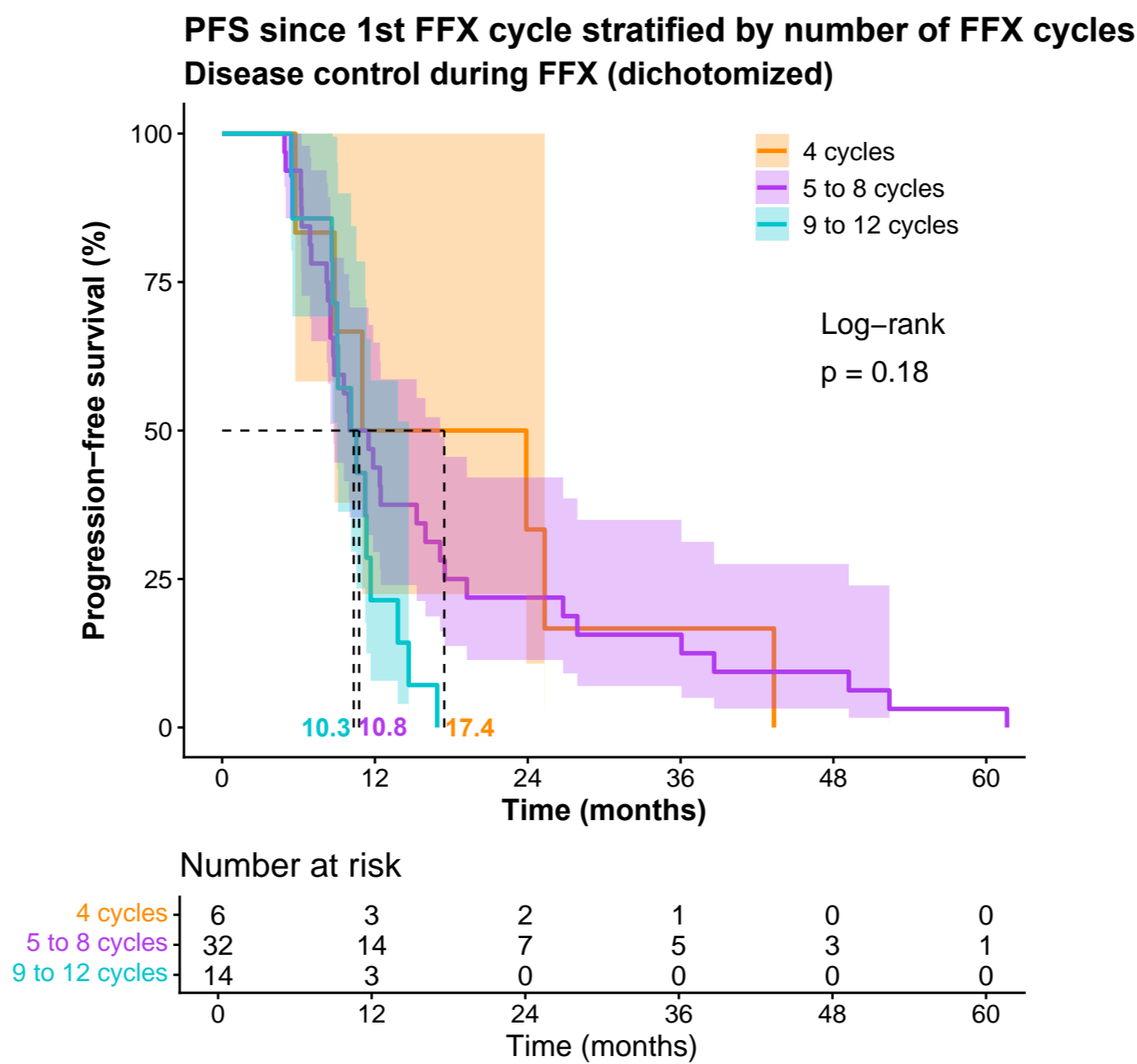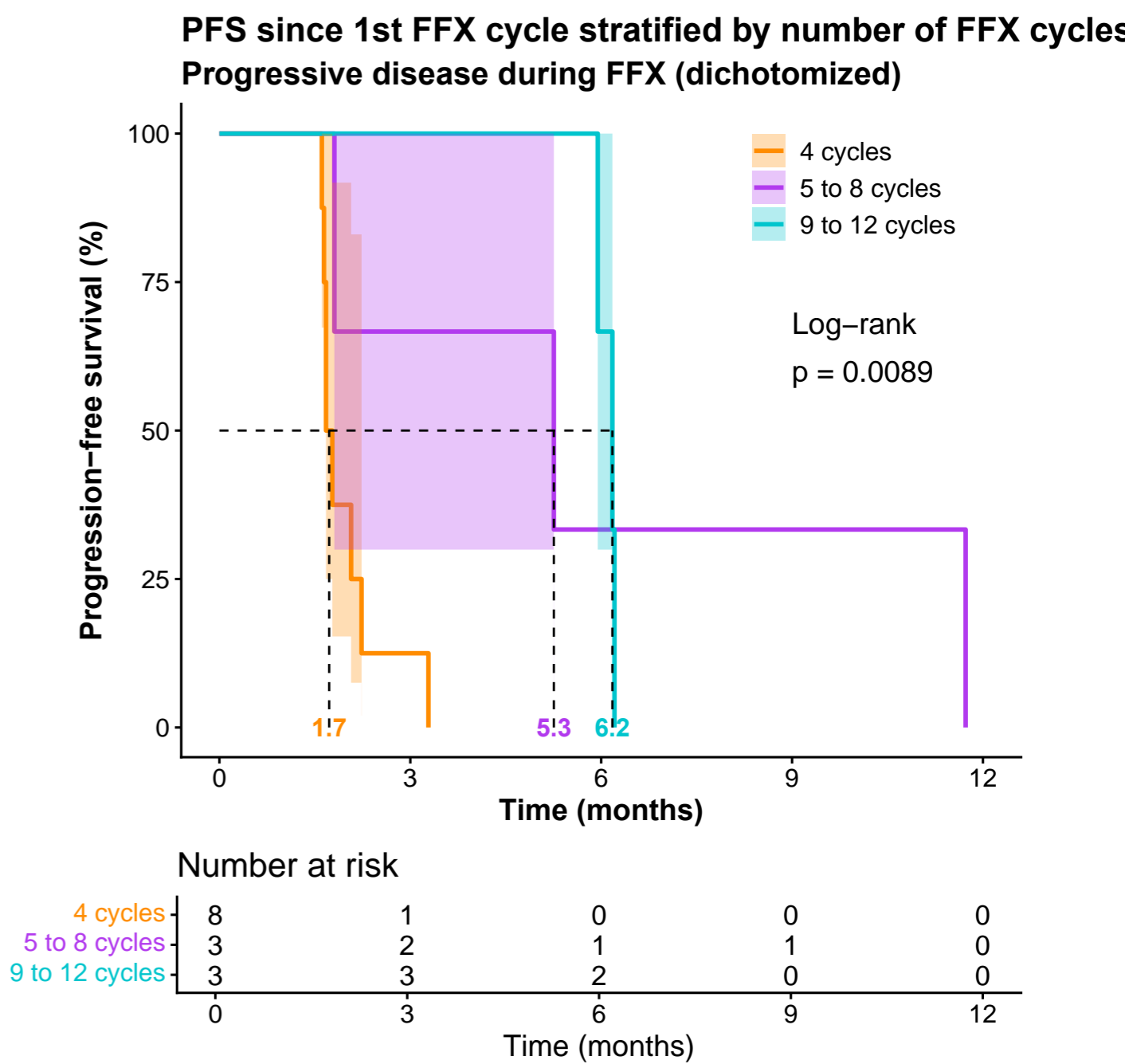

Supplement: Supplementary file 1 — (PDF 93.1 KB) [file 520_2026_10568_MOESM1_ESM.pdf]
